# Heartworm adulticide treatment: a tropical perspective

**DOI:** 10.1186/s13071-023-05690-8

**Published:** 2023-04-28

**Authors:** Filipe Dantas-Torres, Jennifer Ketzis, Gabriela Pérez Tort, Andrei Daniel Mihalca, Gad Baneth, Domenico Otranto, Malaika Watanabe, Bui Khanh Linh, Tawin Inpankaew, Pablo Borrás, Sangaran Arumugam, Barend Louis Penzhorn, Adrian Patalinghug Ybañez, Peter Irwin, Rebecca J. Traub

**Affiliations:** 1 Instituto Aggeu Magalhães, Fundação Oswaldo Cruz (Fiocruz), Recife, Pernambuco Brazil; 2 Ross University School of Veterinary Medicine, Basseterre, Saint Kitts and Nevis; 3 University of Buenos Aires and Virreyes Veterinary Hospital, Buenos Aires, Argentina; 4 University of Agricultural Sciences and Veterinary Medicine of Cluj-Napoca, Cluj-Napoca, Romania; 5 The Hebrew University of Jerusalem, Rehovot, Israel; 6 University of Bari, Valenzano, Bari Italy; 7 University Putra Malaysia, Serdang, Selangor Malaysia; 8 Vietnam National University of Agriculture, Hanoi, Vietnam; 9 Kasetsart University, Bangkok, Thailand; 10 Administración Nacional de Laboratorios e Institutos de Salud Dr. Carlos G. Malbrán, Buenos Aires, Argentina; 11 Madras Veterinary College, Tamil Nadu Veterinary and Animal Sciences University, Chennai, India; 12 University of Pretoria, Pretoria, South Africa; 13 Cebu Technological University, Cebu, Philippines; 14 Murdoch University, Perth, Australia; 15 Melbourne Veterinary School, University of Melbourne, Parkville, VIC Australia

**Keywords:** *Dirofilaria immitis*, Mosquitoes, Melarsomine, Moxidectin, Ivermectin, Doxycycline, Prevention, One health

## Abstract

*Dirofilaria immitis* (the canine heartworm) is widespread in the tropics, with prevalence surpassing 30% in high-risk areas. In addition to the suitable climatic conditions that favour mosquito abundance and filarial larva development, there is low compliance with the recommended year-round use of preventives in these transmission hotspots. This represents a major concern, considering that melarsomine (first-line heartworm adulticide) is unavailable in several tropical countries, resulting in the so-called slow-kill protocol being the only available adulticide treatment option. In this article, the members of TroCCAP (Tropical Council for Companion Animal Parasites) review the current distribution of heartworm in the tropics and the availability of melarsomine, and discuss alternatives for the management of heartworm infections in dogs.

**Graphical Abstract:**

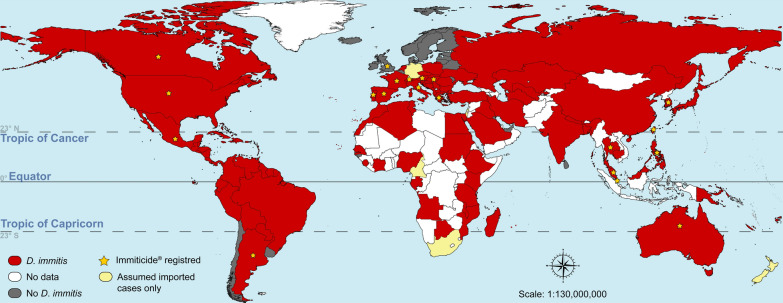

## Background

*Dirofilaria immitis* (the canine heartworm) is a filarial parasite of dogs, which may also infect other hosts, including cats, humans and various wildlife species [1]. This parasite is transmitted biologically by mosquitoes (Culicidae) of several genera and species [2]. When an infected female mosquito takes a blood meal from a host, the tip of her labium ruptures, and a droplet of haemolymph containing infective larvae is expelled onto the surface of the host’s skin [3]. The infective larvae then actively invade the host’s body through the mosquito bite wound and start their journey to the right heart and pulmonary arteries, where they reach sexual maturity approximately 120 days after infection [1, 3–5]. Fertilized females release microfilariae in the bloodstream, allowing them to be ingested by another blood-feeding female mosquito to continue their journey.

Knowledge of the biology of *D. immitis* in dogs and mosquitoes obtained through research in the past decades was instrumental for the development of protocols and strategies to prevent and treat heartworm infections in dogs. Nonetheless, in spite of the scientific advancements, treatment and preventive options are not equally accessible in all regions of the world. An emblematic example is the limited availability of melarsomine dihydrochloride (first-line heartworm adulticide) in many tropical countries where *D. immitis* is highly prevalent. As a consequence, the so-called slow-kill protocol has been widely used in countries where melarsomine is not available.

In the present article, we, the members of TroCCAP (Tropical Council for Companion Animal Parasites), review the current distribution of heartworm in the tropics and the availability of melarsomine, and discuss alternatives for the management of heartworm infections in dogs.

### Distribution, prevalence and incidence of *D. immitis* in the tropics

**Fig. 1:**
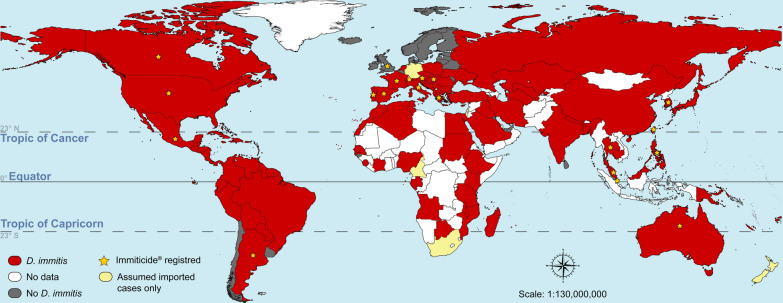
World distribution of *Dirofilaria immitis*. This map was built using QGIS and publicly available shapefiles [84]. Sources of information include several review and research papers [1, 6, 8–27, 31, 32, 34, 35]. The red colour does not mean that *D. immitis* is present in the whole country, particularly for countries lying outside the tropics. Countries where Immiticide^®^ (Boehringer Ingelheim) is currently registered are indicated with a yellow star. In some countries (e.g. United States), melarsomine may also be available as Diroban™ (Zoetis)

The distribution of *D. immitis* in a given area depends on several factors. Climate is a determinant factor, not only for mosquito procreation, but also for the development of infective larvae in the mosquito vector [6, 7]. The tropics present all the necessary elements for the perfect storm: a suitable climate, high abundance of mosquito vectors, large populations of reservoirs in the form of stray or community dogs, and low compliance with year-round use of preventives by pet owners. It is not by chance that *D. immitis* is widespread and prevalent in the tropics [1, 8–10]. The presence of *D. immitis* requires confirmation in many tropical countries (Fig. 1), probably due to the lack of local studies or the lack of publication of these studies in indexed journals. In fact, *D. immitis* is likely present in many countries in Africa, the Middle East and Asia–Pacific, for which there is currently no information in the international literature.

Comprehensive reviews on the distribution and prevalence of *D. immitis* worldwide are available elsewhere [1, 8, 10]. Among the major geographical regions within the tropics (i.e. Central America, Caribbean, South America, North Africa, Middle East, sub-Saharan Africa, South Asia, South-East Asia, East Asia, the Pacific Islands, Northern Australia), the overall prevalence of heartworm in dogs appears to be higher in South America, Central America and the Caribbean [1, 8–11]. Still, the prevalence varies widely across countries, regions, states, cities and even neighbourhoods [9, 11–19]. As examples, Bader et al. [16] tested 945 dogs from three departments in Guatemala and reported an overall prevalence of 29.7%. However, the prevalence varied from 0 to 59.7% among the departments investigated, with the highest prevalence being found in Santa Rosa (south-east coast of Guatemala), where the tropical savanna climate probably favours mosquitoes and *D. immitis* [16]. Similarly, several heartworm transmission hotspots have been detected in the Atlantic coast of Brazil, with prevalence frequently surpassing 30% [14, 15, 20]. This phenomenon is also common in Asia. For example, in Cambodia’s northern province of Preah Vihear, DNA of *D. immitis* was found circulating in 15.8% of 101 community dogs [21], compared to none of 467 community dogs tested by antigen enzyme-linked immunosorbent assay (ELISA) in areas spanning from Siem Reap to Phnom Penh [22]. In South Asia, although *D. immitis* is endemic to Bangladesh [23] and the northern half of India, autochthonous cases of heartworm have not been reported in India’s south [24, 25] or in Sri Lanka [26]. In coastal Queensland, Australia, heartworm has re-emerged in the last decade, with 9.6% of 166 shelter dogs testing antigen-positive [27].

An important aspect to be considered while interpreting data from cross-sectional studies is the diagnostic techniques employed to detect *Dirofilaria* spp. Currently available antigen tests are highly specific for *D. immitis*, although cross-reaction with the parasite *Spirocerca lupi* has been reported [28]. While the sensitivity of antigen tests is relatively high, studies that rely solely on them can underestimate prevalence in cases of single-sex infections or when there is antibody–antigen binding. Heat treatment of serum prior to antigen testing can increase sensitivity [29], but may result in decreased specificity in areas where *Dirofilaira repens* is co-endemic [30].

Studies that rely on the presence of microfilariae can under- and overestimate prevalence. Underestimation occurs when there are occult infections or low numbers of microfilaria (including those related to the circadian variation in the blood), while overestimation occurs when other blood-dwelling microfilariae found in tropical regions are misidentified as *D. immitis*. For instance, a study carried out in Sri Lanka reported the presence of microfilariae in 77 out of 162 (47.5%) canine blood samples examined by direct smear [26]. By polymerase chain reaction (PCR), 95 of these dogs were positive for *D. repens* and none of them was positive for *D. immitis* [26]. This study exemplifies that data from studies reporting microfilaria detection should be interpreted with caution. Another filarial parasite widespread in the tropics is *Acanthocheilonema reconditum* and, to a lesser extent, *Acanthocheilonema dracunculoides* [9, 24, 31–34]. *Dirofilaria immitis* may also coexist with *Brugia pahangi* and *Brugia malayi*, as demonstrated in Thailand [35]. Overall, these filarial species can be identified by detailed morphometric analysis or by molecular methods [31–38].

There is limited information about the annual incidence of heartworm infections in dogs, especially in the tropics. This knowledge gap results from the low number of longitudinal field studies conducted in the tropics, which may be partly explained by the limited investment in research. One should also consider the inherent difficulties in following up a defined population of dogs, especially if they are semi-restricted, with frequent access to the streets without human supervision. In a longitudinal study conducted in the Atlantic coast of north-eastern Brazil, where *D. immitis* is known to be highly prevalent [15], 204 dogs were followed up for 12 months, and the year-crude incidence of heartworm infection was 39.8% (95% CI 30.4–50.0%) [20]. Considering an estimated local population of ~ 3500 dogs, approximately 1393 new infections would occur each year only in this city. This number is alarming, as many cities on the Atlantic coast of Brazil have similar features in terms of climate, landscape and low compliance with heartworm prevention practices.

The widespread distribution and oftentimes high prevalence of *D. immitis* in dogs imply that a number of infected dogs are expected to visit a veterinary practice at some point in their lives. These infections may go unnoticed if the dog is asymptomatic, but many of them will be diagnosed and treatment options considered.

### Heartworm adulticide treatment

The treatment of mature heartworm infections is onerous for veterinary practitioners, owners and dogs themselves. The main reasons are the high cost of treatment, the potentially dangerous side effects and the need for exercise restriction [39, 40]. The American Heartworm Society (AHS)-recommended treatment protocol (last updated in 2020) includes a combination of doxycycline, a macrocyclic lactone and melarsomine (three-injection regimen) (for more details, see [41]). This treatment protocol was endorsed by the European Scientific Counsel Companion Animal Parasites (ESCCAP) [42] and TroCCAP [43]. The Companion Animal Parasite Council (CAPC) and the European Society of Dirofilariosis and Angiostrongylosis (ESDA) differ slightly in their treatment recommendations from the AHS [44, 45], but they both emphasize that the three-injection regimen is the treatment of choice for all heartworm-infected dogs. In addition, they recognize the impact of doxycycline on improved outcome in treated dogs, but they do not recommend a specific regimen for its use [44, 45].

Protocol variations aside, the AHS [41], ESCCAP [42], TroCCAP [43], ESDA [44] and CAPC [45] recommend melarsomine as the first-line heartworm adulticide. In some countries, melarsomine is available in two branded products: Immiticide® (Boehringer Ingelheim) and Diroban™ (Zoetis). However, this drug is unavailable in several countries where heartworm is endemic, as discussed in the next section of this article.

### Limited availability of melarsomine

Melarsomine is not registered in most countries where heartworm disease is endemic (Fig. 1). Moreover, even in countries where it is currently registered, sporadic periods of melarsomine shortage have been reported [39, 40].

The unavailability of melarsomine may be due to varying reasons, including lack of interest from pharmaceutical companies owing to poor market incentive or due to the bureaucratic and lengthy registration processes experienced in some countries. Additionally, some countries have banned the importation, manufacturing, registration, marketing and use of veterinary products containing arsenic or arsenical compounds, as in the case for Colombia [46].

The limited availability of melarsomine in the tropics has numerous consequences for dogs, owners and veterinary practitioners, especially in areas where there is low compliance with the recommendation of year-round use of preventives. The point is: how should veterinary practitioners manage heartworm-infected dogs in the absence of melarsomine?

### The “slow-kill” protocol

The so-called slow-kill or soft-kill protocol includes a combination of doxycycline (or minocycline) for 28–30 days and a heartworm preventive (monthly), such as ivermectin or moxidectin [40, 47, 48]. Doxycycline kills *Wolbachia*, a bacterial endosymbiont that is fundamental for development, reproduction and adult heartworm survival [49]. It also mitigates pulmonary pathology and reduces post-treatment complications [40, 49]. Macrocyclic lactones kill different developmental stages of *D. immitis*, including microfilariae, L3 and young L4 [50]. They are also adulticidal when combined with doxycycline [49].

Pros and cons of the slow-kill protocol have been discussed elsewhere [40, 49, 51]. Initial concerns regarding the lack of efficacy and safety of slow-kill protocols have been demystified by recent studies. An experimental study evaluated the efficacy of topical moxidectin (2.5 mg/kg) with imidacloprid (10 mg/kg) applied monthly for 10 months in addition to doxycycline (10 mg/kg by mouth every 12 h [PO q 12 h] × 30 days) [47]. Efficacy of 95.9% in eliminating adult heartworms was calculated after 10 months [47]. The authors concluded that this protocol is a relatively quick, reliable and safe option to treat heartworm infection in dogs.

In fact, numerous studies using variations of the slow-kill protocol have confirmed their safety and efficacy [40, 47, 52–58]. Studies have demonstrated a greater adulticide efficacy of moxidectin [47, 52–57] as compared to ivermectin [59–61]. This is possibly a result of the unique pharmacokinetics and pharmacodynamics of moxidectin, i.e., it is more lipophilic, with a larger volume of distribution, longer half-life and slower elimination, as compared to ivermectin [40, 62–64].

A recent critical assessment of published slow-kill protocols concluded that a combination of moxidectin (topical formulation, at recommended dose, monthly until no antigen detected) and doxycycline (10 mg/kg PO q 12 or 24 h for 28 days) is an accessible alternative, in cases where melarsomine is unavailable or contraindicated [40]. Dogs under treatment should be tested 6 and/or 12 months after treatment initiation, and treatment success is presumed when the dog is negative to both heartworm antigens and microfilariae. If after 12 months the dog is still antigen-positive, doxycycline therapy should be repeated [40]. A recent study suggested that the detection of *Wolbachia* DNA could be an additional tool to evaluate the therapeutic success of slow-kill protocols [58]. Treatment failure should be considered when a dog is still antigen-positive after > 24 months [40]. Clinical pulmonary thromboembolism seems to be rare, and there is insufficient data to recommend a specific duration of exercise restriction during non-arsenical treatment [40]. The general recommendation is that exercise should be restricted as much as possible until no adult antigen is detected [40].

The benefits of this combination therapy are also recognized in the AHS-recommended treatment protocol, which includes the use of a heartworm preventive (days 1, 30, 61, 90, 120, then year-round, as needed) and doxycycline at 10 mg/kg q 12 h (days 1–28) prior to the three-injection regimen of melarsomine (one injection of 2.5 mg/kg followed at least 1 month later by two injections of the same dose 24 h apart) [41]. Furthermore, the AHS guidelines state that where melarsomine treatment is not possible or is contraindicated, treatment with the slow-kill protocol can be considered as a “salvage” procedure [41]. Jacobson and DiGangi [40] discussed the use of this term and contended that non-arsenical treatment is a reasonable alternative to melarsomine in specific circumstances and should be presented as such. In an electronic survey distributed to the AHS and Association of Shelter Veterinarians' mailing lists, 16.8% of 242 respondents reported the use of non-arsenical treatment protocols in the USA [65].

The slow-kill protocol is not currently recommended as the first-line treatment by the AHS [41], ESDA [44] or CAPC [45], and is not addressed in the ESCCAP guidelines [42]. We, the members of TroCCAP, endorsed the use of melarsomine (three-dose regimen) as the first-line treatment, where melarsomine treatment is available and indicated [43]. When melarsomine is unavailable or contraindicated or in cases where the client cannot afford higher costs associated with melarsomine treatment, the slow-kill protocol may be an accessible alternative [40].

Concerns regarding adulticide heartworm treatment with moxidectin and doxycycline have been extensively discussed and summarized elsewhere [40]. In brief, concerns include progression of pulmonary pathology, exercise restriction, compliance with monthly medication, maintenance of a reservoir of infection, selection for microfilarial resistance and costs [40]. The slow-kill protocol causes less harm than no treatment at all, and pulmonary pathology is reduced by doxycycline. Serious complications are rare even without exercise restriction. The compliance issue is similar for preventives, and sustained-release formulations could offer a solution. Regarding the reservoir status, while adults take longer to die, moxidectin rapidly clears microfilariae, breaking the transmission cycle [58]. The concern regarding selection for microfilarial resistance also exists with year-round preventives, and evidence suggests that moxidectin may be effective against resistant strains. Moreover, even if microfilariae do not die, they become non-infective [61, 66, 67]. In terms of cost, the long duration of the slow-kill protocol may raise the total cost of treatment, but it is still less expensive than the full protocol, which is important in the tropical context.

Regardless of the protocol used, slow-kill or the full AHS protocol, it is important to consider that not all dogs tolerate doxycycline or minocycline [48]. The frequency and severity of gastrointestinal side effects (e.g. vomiting and diarrhoea) were greater in dogs receiving 10 mg/kg q 12 h of either drug compared to 5 mg/kg q 12 h in a study from 2018 [48]. There was no significant difference in the rate of microfilarial clearance between groups treated with doxycycline or minocycline, regardless of dosage [48]. The authors recommended that veterinarians should prescribe 10 mg/kg q 12 h of doxycycline for 28 days and, in cases of severe gastrointestinal side effects, reduce to 5 mg/kg q 12 h [48]. The use of doxycycline once daily (10–20 mg/kg q 24 h) instead of twice daily (5–10 mg/kg q 12 h) may help increase owner compliance with treatment recommendations.

Another primary concern regarding heartworm adulticide treatment is the overuse of antibiotics, especially ones critical to human medicine. Doxycycline is the primary treatment for several human diseases, such as Lyme borreliosis, spotted fever rickettsioses, scrub and murine typhus, and filariases [68, 69], as well as for canine monocytic ehrlichiosis (*Ehrlichia canis*), canine cyclic thrombocytopenia (*Anaplasma platys*) and canine granulocytic anaplasmosis (*Anaplasma phagocytophilum*) [70–72]. While there is no evidence that the use of doxycycline for heartworm treatment in dogs changes the efficacy against these critical diseases in people, this concern deserves further research.

### Why treatment of heartworms in the tropics matters?

Heartworm infections can be life-threatening and should be treated as early as possible. Indeed, annual screening may help in detecting early infections and allow veterinary practitioners to start treatment even before the appearance of clinical signs. In this initial phase, most dogs will go through adulticide therapy without complication [51]. Advanced heartworm disease may be more difficult to treat and may include severe manifestations such as heart failure and caval syndrome, which may prove fatal [51]. In a study carried out in Grenada, heartworm infection was found in 249 out of 1617 dogs necropsied from 2001 to 2013 at the School of Veterinary Medicine, St. George’s University [73]. Among the 249 infected dogs, 33 (13.2%) had caval syndrome.

The treatment of heartworm infections should also be seen from a one heath perspective. In fact, in addition to being life-threatening for dogs, *D. immitis* is zoonotic [1, 74–76]. While human dirofilariosis caused by *D. immitis* is often regarded as a rare zoonosis [77], there is limited information on the level of human exposure to heartworm in the tropics, which may well be underestimated considering the level of exposure in dogs and the abundance of competent mosquito vectors. Therefore, the treatment of infected dogs should also be considered important to reduce the reservoir of infection and the zoonotic transmission risk.

Untreated dogs serve as reservoirs of infection for mosquitoes and ultimately other dogs (and people). In this scenario, lack of treatment can become a global issue with the increasing movement of dogs between regions. After hurricane Katrina, dog movement from New Orleans to many other regions of the USA contributed to the spread of heartworm and, more critically, resistant strains of heartworm [78, 79]. The occurrence of natural disasters such as hurricanes are more common in tropical regions, and these events can lead to mass movement of dogs. If treatment solutions are not found locally, then there is an increased risk of movement of heartworm-positive dogs with the introduction of different strains to new locations.

Movement of dogs from highly endemic areas to areas of low endemicity may increase the risk of local heartworm transmission [79]. A study reported a 48.8% positivity in 1958 dogs rescued and relocated from the Gulf Coast following the hurricanes in 2005, as tested by numerous animal welfare groups in 37 different states in the USA and in Alberta, Canada [80]. From a global perspective, the increased movement of dogs around the world could also facilitate the introduction of tropical heartworm strains in temperate countries and vice versa. For instance, roughly 1.06 million dogs are imported into the USA each year [81]. Likewise, thousands of dogs are imported to the UK [82]. This highlights the need to consider the treatment of heartworms in the tropics from a global perspective.

## Conclusions

Heartworm infection is widespread and prevalent in the tropics, and likely underestimated in some regions due to the limited availability of studies published in the international literature, particularly in Africa. There are also no reliable, long-term data to conclude whether heartworm prevalence is increasing or decreasing in the tropics. In fact, premature comparisons of data from different cross-sectional studies may lead to misleading conclusions regarding prevalence trends in different regions. As with any vector-borne disease, the prevalence of heartworm infection in a given region depends on several biotic and abiotic factors. In this regard, long-term longitudinal studies are fundamental to understanding the trends in heartworm prevalence in the tropics. Melarsomine is currently unavailable in most tropical countries, and the slow-kill protocol should be considered as an accessible alternative. Efforts should be made to increase the availability of melarsomine in the tropics, as this drug remains the first-line heartworm adulticide. Finally, improving knowledge about heartworm and its prevention in the tropics is pivotal, as discussed elsewhere [83]. From this perspective, veterinarian and pet owner education is key to increasing compliance with year-round prevention so as to reduce the burden of heartworm disease in these regions.

## Data Availability

Not applicable.
